# Towards Health in All Policies for Childhood Obesity Prevention

**DOI:** 10.1155/2013/632540

**Published:** 2013-04-16

**Authors:** Anna-Marie Hendriks, Stef P. J. Kremers, Jessica S. Gubbels, Hein Raat, Nanne K. de Vries, Maria W. J. Jansen

**Affiliations:** ^1^Academic Collaborative Centre for Public Health Limburg, Regional Public Health Service, P.O. Box 2022, 6160 HA, Geleen, The Netherlands; ^2^Caphri, School of Public Health and Primary Care, Maastricht University, P.O. Box 616, 6200 MD Maastricht, The Netherlands; ^3^Faculty of Health, Medicine and Life Sciences, Department of Health Promotion, Maastricht University, P.O. Box 616, 6200 MD Maastricht, The Netherlands; ^4^NUTRIM, School for Nutrition, Toxicology and Metabolism, Maastricht University, P.O. Box 616, 6200 MD Maastricht, The Netherlands; ^5^Department of Public Health, Erasmus MC University Medical Center Rotterdam, P.O. Box 2040, 3000 CA Rotterdam, The Netherlands; ^6^Faculty of Health, Medicine and Life Sciences, Department of Health Services Research Maastricht University, P.O. Box 616, 6200 MD Maastricht, The Netherlands

## Abstract

The childhood obesity epidemic can be best tackled by means of an integrated approach, which is enabled by integrated public health policies, or *Health in All Policies*. Integrated policies are developed through intersectoral collaboration between local government policy makers from health and nonhealth sectors. Such *intersectoral collaboration* has been proved to be difficult. In this study, we investigated which resources influence intersectoral collaboration. The *behavior change wheel* framework was used to categorize motivation-, capability-, and opportunity-related resources for intersectoral collaboration. In-depth interviews were held with eight officials representing 10 non-health policy sectors within a local government. Results showed that health and non-health policy sectors did not share policy goals, which decreased motivation for intersectoral collaboration. Awareness of the linkage between health and nonhealth policy sectors was limited, and management was not involved in creating such awareness, which reduced the capability for intersectoral collaboration. Insufficient organizational resources and structures reduced opportunities for intersectoral collaboration. To stimulate intersectoral collaboration to prevent childhood obesity, we recommend that public health professionals should reframe health goals in the terminology of nonhealth policy sectors, that municipal department managers should increase awareness of public health in non-health policy sectors, and that flatter organizational structures should be established.

## 1. Introduction

Childhood obesity is currently considered an epidemic. Prevalence rates have doubled over the last three decades. Globally, approximately 180 million children (<18 years) are estimated to be overweight or obese [1–3]. In 2010, 43 million of them were under the age of five [3]. This rapid development has focused much attention on the problem (e.g., [4, 5]), especially since childhood obesity is associated with many health problems [6]; it often tracks into adulthood [7] and causes huge rises in health care costs [8]. 

The childhood obesity epidemic shows predictable patterns in almost all countries, due to similar systemic drivers (policies and economic systems) and environmental drivers (marketing of energy-dense foods and facilitation of passive transport) promoting overconsumption and physical inactivity [4]. Interaction between individual factors (e.g., genetic predispositions) and the environments in which children grow up (e.g., their neighborhoods) lead to behaviors that cause a positive energy balance and in the long run weight gain [9]. In view of these drivers and the related economic and public health consequences of obesity, many experts have stressed the need for governments to take action (e.g., [10]). 

Since it is recognized that health, and specifically obesity, is influenced by determinants not only within the health domain, but also outside this domain, experts recommend the implementation of an “integrated approach” for this so-called “wicked problem” [11–14]. An integrated approach is characterized by a mixture of coordinated interventions and policies by multiple disciplines, organizations, and sectors. Integrated public health policies, often referred to as “*Health in All Policies*” (HiAPs), are an important part of any integrated approach since they enable its implementation [13]. The HiAP approach is defined as: “*a horizontal, complementary policy-related strategy with a high potential for contributing to population health*” [11, 12]. The terms “horizontal” and “complementary” refer to the distinguishing feature of “integrated” compared to “regular” health policies, namely, intersectoral collaboration. Ensuring that health is taken into account in policies that are developed in other policy sectors requires close collaboration with these other sectors within government; thus, intersectoral collaboration is a prerequisite for the development of integrated public health policies [13]. An example of such an integrated policy developed through intersectoral collaboration is the policy to encourage active transport by improving road safety (collaboration between the public health and transport sectors). The implementation of such policies has been proved to be difficult; barriers are inherent to the *intersectoral* as opposed to *intersectoral* character of the collaboration and thus hamper the development of integrated public health policies [15–18]. Moreover, research shows that intersectoral collaboration within local governments is rarely established, and attempts to explore which factors cause this lack of collaboration have been scarce [19]. Some studies suggest that factors related to the topic of “childhood obesity prevention” (content-related factors) are responsible for the lack of intersectoral collaboration, while other studies suggest factors related to the process of intersectoral collaboration (process-related factors).  Table 1 lists examples of these factors, based on an exploration of the literature. The literature review did not aim to be exhaustive but to provide a panoramic view of possible barriers and facilitators.

A limitation of these studies is that most of them were conducted in the context of organizations (e.g., focusing on interorganizational relationships) or community coalitions, and few specifically focused on the development of integrated public health policies to prevent childhood obesity within local governments. Therefore, this study focused on the resources (i.e., facilitators and barriers) regarding intersectoral collaboration for public health in general, and for childhood obesity prevention specifically. 

**Figure 1:**
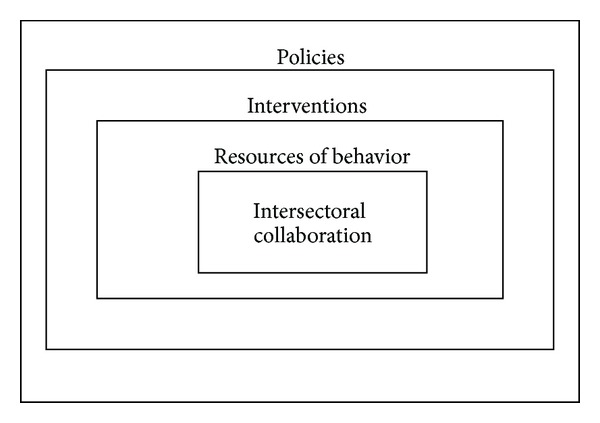
The behavior change wheel, adapted from Michie et al. [21].

To capture the resources needed for intersectoral collaboration and the development of integrated public health policies, we have used the “behavior change wheel” framework developed by Michie et al. [20, 21] (Figure 1). We adopted this framework since it provides a clear structure for reflecting upon resources for intersectoral collaboration and thus could help us answer our research question. 

The framework is based on the idea that behavior is determined by the following three resources: motivation, capability, and opportunity. If one of these resources is lacking or insufficiently present, behavior change interventions, which can be implemented by certain policies or programs (the outermost circle), might be needed to increase the likelihood of achieving intersectoral collaboration [20–22]. The present study focuses on the resources for intersectoral collaboration, as described below. 

*Motivation* can be divided into reflective and automatic processes. Reflective motivation involves more conscious decision-making in evaluations and plans [21]. An example is having positive beliefs about the outcomes of intersectoral collaboration. Automatic motivation is based on emotions and impulses that arise from associative learning or innate dispositions. An example of automatic motivation is experiencing work engagement [48]. 

*Capability* is the extent to which individuals can adapt to change, generate new knowledge, and continue to improve their performance [49]: “*capability is what people are able to do and to be*” [50]. Psychological capability refers to the capability to engage in the necessary thought processes such as comprehension and reasoning [21], and it is closely related to competence, which refers to what individuals know or are able to do [49]. An example of psychological capability is having boundary-spanning skills [51].

*Opportunity* refers to conditions that are external to the individual actor. Two forms of opportunity are distinguished: physical and social. Physical opportunity is afforded by the working environment (e.g., organizational structures), while social opportunity refers to the municipal situation that dictates the way people think about things, the words and concepts they use, and the predominant discourse (e.g., organizational culture) [21]. 

The current dearth of knowledge regarding factors that facilitate or hamper intersectoral collaboration within local governments might explain why integrated public health policies have not been frequently applied in practice. Therefore, we aimed to answer the following research question: *What resources do local nonhealth policy makers need in order to collaborate with the health sector in the prevention of childhood obesity?*

## 2. Methods

### 2.1. Study Sample and Design

 In this study, we used a single-case study design [52] and in-depth semistructured interviews to collect our data. Our study sample consisted of eight policy officials working in a Dutch municipal government, responsible for 10 different policy sectors (some officials being responsible for more than one sector) (Table 2). At the time of the interviews (summer 2011), this municipal government employed 65 people. The municipality has around 11,000 inhabitants and covers an area of about 16 km^2^. 

Two interviewers jointly conducted all interviews: one (male) public health official working at the municipal government in which this study took place and one (female) university researcher. Afterwards, the two interviewers reflected on each of the interviews to compare notes and arrive at a more accurate interpretation of the data. Their reflections were entered into the reports that were sent afterwards to each of the interviewees and were also used in the data analysis. The interview reports were sent to the interviewees by way of member checks, in order to increase the reliability of our data. The interview protocol was jointly defined by the two interviewers (see appendix). Throughout the interview, we used childhood obesity prevention as an example of a public health problem that could be addressed more effectively if health and nonhealth sectors would collaborate. Our approach aim was to first focus on intersectoral collaboration for public health *in general* and then to focus on the prevention of childhood obesity. We assumed that this approach would reveal more information about resources for intersectoral collaboration than narrowing down our focus too early. Furthermore, we assumed that resources for intersectoral collaboration would be comparable, as long as the policy issues that were being discussed had a “wicked” character [34].

### 2.2. Data Analysis

 The university researcher transcribed the relevant parts of the interviews, categorizing them under subheadings that were based on our predefined interview items. She then sent the resulting reports to the public health official (the second interviewer), who sent them to the interviewees for a final accuracy check. Thus, the public health official was not only one of the interviewers, but he also assisted the researcher in conducting member checks. The interviewees were asked to send any comments to both interviewers. When the interviewees made any comments, the researcher checked and adjusted the transcripts and sent them once more to the public health official (the second interviewer). This approach ensured the accuracy of the transcripts. The transcripts were analyzed with the help of NVivo software, using the behavior change wheel as the theoretical framework to code the responses [21]. 

## 3. Results 

Based on our analysis, we describe here the facilitators and barriers regarding intersectoral collaboration that we identified. Each facilitator or barrier is categorized under the factors of motivation, capability, or opportunity. For each quotation, we state whether the interviewee was male (M) or female (F) and the department for which they worked.

### 3.1. Motivation

 The main perceived barrier to intersectoral collaboration that was mentioned during the interviews was that the health and nonhealth sectors did not have the same policy goals. This could reduce the motivation among non-health policy makers to involve the public health sector in an early stage of policy development and take aspects relevant to public health into account. Health was perceived as a side issue, as was expressed by a spatial planning official:

* “Those are all side issues, as you start with a different goal in mind. Somebody comes along and says … I want to build a house, and it's only then that you start thinking, and then it's a matter of getting a house built there … You can come up with other things as you go along. … But our goal is not public health*.” (F, spatial planning official.)

Nevertheless, policy goals were frequently similar, even if officials were not aware of this. For example, in the case of creating activity-friendly environments, similarities of policy goals were discovered during the interview; the transport department official commented how one of his policy goals, creating safe roads to walk and cycle on, had a positive effect on the residents' level of physical activity and thus affected public health:

* “The idea of “sustainably safe” actually means that you try to design neighborhoods with that concept in mind, in other words, a 30* *kilometers an hour speed limit.”* (M, transport department official.)

Asthere were such similarities between policy goals, this facilitated intersectoral collaboration. However, most respondents seemed surprised, as they were not aware of the similarities, since they had never explicitly incorporated health in their policies. For example, the official responsible for youth services realized that she had not been paying attention to public health themes in her work routines, which she could easily have done:

*“In children's and youth services you could … select themes that relate to health and overweight prevention.”* (F, official for youth services, social services and tourism.)

Not only the health sector, but also other policy sectors with less dominant policy frames, such as that of municipal environment and tourism indicated that they tended to be “forgotten” when a new policy was being developed. For example, when a new residential area is being designed, the municipal environmental department was not involved until the project was nearly complete:

* “Initiatives for construction work are first presented to the spatial planning department. … and they look mostly at the planning aspects. … So certain things tend to be overlooked at first.”* (M, municipal environment official.)

Another barrier to collaboration with the health sector was the difficulty of making health goals visible and measurable. This appeared to cause stereotyping of the health sectors as being “soft” and “more interested in talking than doing,” while nonhealth sectors (especially the more technically and construction-oriented departments) achieved “real” (visible and measurable) results. The stereotyped perceptions of the representatives of the various sectors were seen as an obstacle to intersectoral collaboration. In line with this, respondents emphasized that health and non-health sectors have different “world perspectives.” According to the interviewees from the “welfare-oriented” sectors (i.e., policy sectors with the primary goal of increasing the subjective well-being of the citizens), the “technically oriented” sectors (i.e., policy sectors with the primary goal of improving the physical environment of the citizens) think health is important in life, but only after economic targets have been met:

* “They look at certain things in a different way, they're people who have a very different background, different training. It's the sector of hard facts. They're concerned with money, bricks and mortar, they just have a different perspective.* (M, public health official and second interviewer.)

* “they might exaggerate and say “You just talk about all kinds of stuff”, and we would say “You never think about people”.”* (F, official for youth services, social services and tourism.)

It was striking that the technically oriented sectors themselves were quite positive about taking health into account. They just framed their policy goals differently; instead of emphasizing health outcomes, they used terms like “aging in place” (*levensloopbestendig*), “sustainably safe” (*duurzaam veilig*), “balanced” (*in evenwicht houden*), or “livability” (*leefbaarheid*) to express their views on preferred outcomes. One respondent referred to developments in a new residential area and their potential positive effects on public health:

* “You can achieve that. … What you do take into account is whether it enables people to “age in place”.”* (F, spatial planning official.)

Most respondents mentioned the territorial attitude of some policy makers; they defend their own work domain and do not allow others to get involved in their professional work, on principle. The extent of this territorial attitude also depends on people's individual character; the main personal factors that were mentioned were whether people trust their colleagues (i.e., feel it is safe to approach other policy actors) and whether they have an open personality (being positive about change, being receptive to new experiences):

* “It's often a matter of character … people with a background in technology take a different view on people … they have very different characters.” *(F, official for youth services, social services, and tourism.)

### 3.2. Capability

One of the barriers within the “capability” category was the lack of knowledge about the nature of public health:

* “There's not a great deal of knowledge about health among the local authorities. … It's certainly not a bad idea to involve the regional Public Health Service” *(M, municipal environment official.)

Understanding how health should be taken into account and the importance of taking it into account was a “new” way of thinking for many non-health policy sectors. During the interview, one official from the spatial planning department admitted that she had always reduced public health to the presence or absence of illness rather than aspects like healthy lifestyle:

* “We simply do not think about that. To me, public health is simply something like whether people get ill or not, and you do not build houses with that in mind.”* (F, spatial planning official.)

To many municipal officials, and especially those with a non-health professional background, public health is a very “abstract” concept, which is not very visible to them. Non-health policy actors therefore frequently proposed to make the concept of public health more concrete. This could also improve the ability of non-health sectors to relate the outcomes of their own work to public health outcomes or to use public health as a vehicle to achieve their own policy goals (or the other way round). When talking about the influence of spatial planning on public health, both of the spatial planning officials we interviewed referred to their lack of awareness of ways they could improve public health. For example, both referred to their own policy themes, such as developing attractive green spaces and water features to improve the esthetics of the landscape, but they did not know this could also improve public health by encouraging people to go walking or cycling more often:

* “Whether we do this consciously, I do not think so.” *(F, spatial planning official.)

*There are many things that you take care of, but without saying so. You incorporate those themes [i.e., themes that can affect public health] in your town planning designs* … (M, spatial planning official.)

In this context, the leading role of the heads of departments was also mentioned by one of the respondents; they should check whether policy proposals are integrated

* “That would be my advice, that they should at least ensure that.”* (Official for youth services, social services, and tourism.)

### 3.3. Opportunity

 Facilitators in the opportunity category included the availability of sufficient resources (e.g., time, money, and policy free space) to adjust policy plans to ensure public health outcomes, and the recognition that citizens require facilities that promote health (recognizing that it is in the interest of citizens that municipal authorities pay attention to public health). When talking about the actors involved in policy-making, one respondent commented that policies used to be largely developed behind closed doors (by policy makers), but that the role of the public in policy-making has now expanded

* “We listen to people's wishes. If signals come from the public, we try to respond to them. … Citizens have a large say in their residential environment. You see the same in other municipalities.”* (M, spatial planning official.)

In this context, respondents also mentioned the benefits of working within a small municipality as follows: (1) officials from different policy sectors know each other and often work within a short physical distance from one another and thus have close social ties and physical proximity and (2) smaller municipalities were said to be more sensitive to the needs (including public health needs) of their citizens. One respondent referred to the occasional lack of opportunity to take these needs into account (e.g., in developing footpaths and safe crossings):

* “If it does not work out that's usually due to money problems.”* (M, sSpatial planning official.)

Organizational structures were said to hamper intersectoral collaboration since they are organized along sectoral lines. In practice, this meant that several sectors did not share a manager who would be responsible for more than one sector, and who could focus on the elements shared by the sectors. One official referred to the facilitative role of the change that had taken place in the organizational structure of their municipal government (which became flatter as a result of departments being merged) and the effect this had had on the distance and collaboration between policy makers from different sectors:

* “It's only recently, since we're housed together, that we hear each other's views. Until recently, we might write a policy plan here, while the people at spatial planning established a different policy plan that wasn't compatible at all. We're now trying to prevent that in the new department, but I do not even want to think about the way these things go in larger municipalities.”* (Official for youth services, social services, and tourism.)

This official indicated that the bureaucracy in larger municipalities is widening the gaps between policy makers and thus raising the barriers to intersectoral collaboration. 

When talking about the role of organizational structures, respondents also mentioned the difference in agenda-setting in the various policy domains. Organizational structure could lead to convergence or divergence of interests. One official referred to the lack of interest among the technically oriented sectors in maintaining a welfare institution. This hampered the achievement of welfare-oriented policy goals, since the technically oriented sectors were not interested in supporting such an institution:

* “For instance when it's about the use of buildings, we from a welfare point of view think it's important that such a [welfare] institution continues to exist, but [the technically oriented departments] have other interests.* (Official for youth services, social services, and tourism.)

This also relates to the next barrier: budgets as well as responsibilities (and goals) tend to be allocated along sectoral lines and are also related to the relevant “cultural” differences between the various policy sectors:

* “You also notice differences of opinion, especially differences in departmental cultures. For instance I'm also responsible for tourism, and from a tourist perspective I would have preferred a different option [referring to designing attractive sites for tourists], but we weren't involved at that stage. Well and by the time we were informed about it, everything had already been settled.”* (F, official for youth services, social services, and tourism.)

Thus, each policy sector uses a different strategy to achieve their diverging goals. This divergence in policy goals makes it difficult to align strategies:

* “When you look at the current plans, you cannot say we're specifically considering public health… We're not really trying to see whether we can actively, involving the built environment, playground equipment for kids and so on.”* (M, spatial planning official.)

* “It's just not that easy”* (F, spatial planning official.)

There are few opportunities to align policy strategies since less dominant policy departments are systematically being involved in the policy development cycle at too late stage. One respondent said that construction plans were usually first implemented, and his sector was then asked to repair the damage:

* “I get the feeling that if social services had been involved in this at the first planning stage…” * (M, Public health official.)

* “It would have been a completely different plan.”* (F, official for youth services, social services, and tourism.)

* “And would that be intentionally or unintentionally?” * (F, university researcher) >* (both policy officials appear very uncomfortable because it is a sensitive topic.) *

Further barriers that decrease the opportunities to adjust policy plans to public health goals were said to be national standards or legislation, which might hamper the perceived ability to take health aspects into account, since they were sometimes either too strict or too loose. If those national guidelines were not strict, tightening them would improve public health outcomes; this was often difficult since it would affect economic performance or be impossible due to the budget cuts:

* “There are a number of guidelines, and we try to stick to them … as long as the budget allows it. … I find that this year we cannot include any measures for the “sustainably safe” campaign [a concept in which neighborhoods are designed in such a way that they create environments promoting safe active transport] in the operational budget.” * (M, transport department official.)

When non-health policy sectors are not sure of the influence their policy has on health, and they want to be advised on this, they have to pay to obtain such information from the regional Public Health Service:

* “but if it [the question regarding public health advice] is not specific, we have to pay for it.” * (M, municipal environment official.)

In addition to this, there are the budget cuts that municipal governments have had to introduce due to the economic crisis. Maintaining sports facilities requires large sums of money, which are currently difficult to make available. Also, the current neoliberal political climate aims to decrease government involvement in policies on community organizations (fewer regulations):

* “At the moment, we're mostly trying to create the right conditions for sports facilities. … We're not going to tell the clubs what to do [*e.g.,* regulating the availability of healthy snacks in their canteens].”* (M, official for education, child care, sports, and cultural affairs.)

Although governments are less involved in using subsidies to control local organizations, a potential for imposing some controlling requirements was mentioned:

* “We have a number of subsidy schemes [to improve public health] but we do not prescribe what they have to do, their policies … But you could think about that, you could come to agreements with them, like for instance we want you to pay attention to such and such once a year [referring to various health topics]., * (F, official for youth services, social services, and tourism.)

Conservative local organizations (which are unwilling to pay attention to health aspects) can also hamper the implementation of an integrated approach:

*“These clubs, they do not feel the need to organize after-school activities. They still have enough members. Like the idea of taking over gym classes; they do not feel the need.” * (M, official for education, child care, sports, and culture.)

The rigidity of organizations was also mentioned as a factor impeding collaboration. For example, even if management is in favor of collaboration, when those at the operational level do not want to change, it will take a long time before a school or sports clubs actually implement, for example, food policies that take health into account. Therefore, a lot of perseverance was said to be needed on the part of the health sector to get integrated public health policies implemented. Additionally, the commercial nature of most community organizations could reduce the opportunities to implement certain health policies because they might put them at a competitive disadvantage:

* “The first thing people throw away is the greens [*e.g.,* a piece of lettuce and a slice of tomato]. You just find it thrown away somewhere. So then you could say you should not sell fatty snacks, but then they're a commercial enterprise, they have to make a living.” * (M, official for education, child care, sports, and culture.)

## 4. Discussion

This study examined the resources that policy actors from non-health-related government sectors needed in order to collaborate with the health sector in developing integrated public health policies. Our interviews showed that six factors, divided over the three resources of motivation, capability, and opportunity, represented the most salient barriers to intersectoral collaboration. These resources are relevant for the development of integrated public health policies to prevent childhood obesity, but they are thought to be similar for “wicked” public health problems in general. The factors included specific discipline-related policy goals and territoriality (motivation), a disability to relate one's own work to public health and the failure of management to facilitate this (capability), and a lack of resources and inappropriate organizational structures (opportunity). Below, we present some recommendations for each of these resources, which may help to achieve a transformation of the current fragmented situation into one of integration.

### 4.1. Motivation to Collaborate: Bridging Gaps May Not Be as Difficult as It Seems

Firstly, there was little motivation among the non-health departments to collaborate with the health sector, since the non-health departments claimed to have different policy goals than public health. Their goals were related to their own policy discipline and thus hard to change. Each policy domain works on the basis of its own logic and without regard for the impact on other areas of society. Such “disciplinarity” was also found to hamper intersectoral collaboration in the study by Bovill [33]. The non-health sectors do not receive any incentives to collaborate with the health sectors, since they are judged (by management and municipal executives) on the basis of a set of criteria that are specific to their department. Nevertheless, when we asked respondents about the content of these “diverging” policy goals, we found that the goals of most non-health sectors were sometimes clearly related to public health goals, sometimes even to such an extent that they might easily be replaced by public health goals. For example, the Department of Transport said they were highly motivated to make their municipality very safe for cyclists, and that “promoting sustainable environments” was the essence of their work. A “sustainable environment,” however, is almost identical to the public health goal of promoting a “leptogenic” environment, since both terms describe an environment in which citizens feel safe and encouraged to use active means of transport (i.e., cycling, walking). However, this link was overseen by both sectors, and bridging the gap between these disciplines, thus, seemed difficult, while in fact the bridge was already present (it only needed to be detected). 

This barrier to collaboration might be overcome if public health professionals could reframe a health topic in such a way that it matches the terminology of the other policy sectors. Reframing health issues in terms understood by the nonhealth policy sectors can help remove the need to compete with those more dominant policy frames. Such re-framing is especially urgent for childhood obesity, as this is still described as a matter of individual responsibility, so that only a set of limited and mostly ineffective policy strategies to prevent childhood obesity come into view. Therefore, public health professionals need to put effort into understanding the goals and vocabulary of other relevant disciplines, in order to be able to re-frame the debate on childhood obesity in such a way that other policy domains will also realize the risk that childhood obesity poses for the achievement of their own policy goals (e.g., reduced economic performance due to obesity-related work absenteeism). As Stone stated: “*Nothing is a risk until it is judged to be a risk*” [53]. 

Secondly, the more welfare-oriented policy makers reported that territoriality was hampering intersectoral collaboration. This finding is also in line with the research findings reported by Bovill [33]. Territoriality was related to the different “world perspectives” in their different policy domains (i.e., their territories). It was remarkable that only those respondents who were working in policy fields perceived to be more closely related to the public health sector (e.g., youth services and sports) reported that the outlook of the more technically oriented policy sectors (e.g., spatial planning and transport) was fundamentally different from their own. According to them, the lack of visible results of health policies was a key distinctive feature explaining why the policy field could be divided into two “subcultures.” Technical sectors focus on bricks and mortar (i.e., changing the physical environment), while welfare sectors focus on people (i.e., the subjective well-being of citizens). Measuring subjective well-being is clearly much harder than measuring physical changes. In the view of welfare-oriented policy makers, the technically oriented policy makers are stereotyping them as “talkers” rather than “doers.” This attitude was, however, not explicitly confirmed by the statements of the more technically oriented policy makers themselves and thus might represent an unintentional preconception on the part of the welfare sector. 

A way to overcome this territoriality problem is to make health outcomes more visible; increased understanding of each other's work may reduce the stereotyping currently experienced by welfare-oriented policy makers. Additionally, frequent communication can be expected to familiarize policy sectors with one another and increase trust “familiarity breeds trust”, which was also mentioned to be an important facilitating factor for collaboration [54].

### 4.2. Capability to Collaborate: The Blind Leading the Blind

Since policy makers were not used to collaborating with policy sectors outside their own “niche,” their experience of intersectoral collaboration was limited (see also [16, 17, 45]). Most respondents argued that it was “new” for them to think explicitly about public health outcomes in relation to their own work. Although they unconsciously paid attention to public health aspects, such decisions were not consciously made and thus not communicated explicitly to the health sectors. This finding is in line with those by Aarts et al. [23], who found that most policy sectors were in fact paying attention to public health, without being aware of it. 

In line with the suggestions made by R. Axelsson and S. B. Axelsson [46], this barrier can be overcome by increased communication and stimulating joint planning. We recommend more explicit communication about the current (sometimes health-promoting) decisions of nonhealth sectors to increase awareness about the links between the health and non-health sectors. Regional Public Health Services can assist by highlighting the similarities between the work of both sectors. To this end, Public Health Services also need to expand their skills. In addition, sufficient joint planning would enable alignment of policy strategies. Mismatches, which were sometimes so pervasive that certain policy documents (in which much time and effort had been invested) had to be rejected completely, can be prevented through early alignment. One tool that can be used to explore more specific strategies to achieve such alignment is contribution mapping [55]. 

In this context, a fourth barrier was also identified: the failure of the heads of departments to stimulate intersectoral collaboration. Within hierarchical organizations, heads of departments manage the work processes that can lead to intersectoral collaboration, so their potential influence is large (at least in theory). One explanation for the lack of involvement of management might be that, as was found by Steenbakkers et al. [45], managers lack sufficient know-how for intersectoral collaboration. Managers could adopt an ambiguous attitude towards the pursuit of integration because they are aware of the demands this would impose on them. Moreover, their inexperience in this “new” job requirement might make them feel insecure about their own ability to do the job and thus create stress. Another cause of stress might be related to their fear of losing status: within hierarchical organizations, integration requires system-wide changes. Merging several departments requires changing organizational subcultures into one new organizational culture, and these cultural changes should be complemented by changes in the organizational structure to be sustainable. By making managers responsible for more than one sector, they might become more focused on the elements shared by the various sectors. However, this requires changes that can put the status of actors higher up in the hierarchy into question. The expectation of losing status might reduce the motivation among management and municipal executives more than among operational level actors (the higher in the hierarchy the more power they stand to lose). To keep the system as it is, higher level actors might therefore intentionally inhibit real changes (i.e., changes that might be truly effective for intersectoral collaboration). Previous studies [56] have identified that, within local governments, process management is insufficiently implemented and a more central role of “liaison” manager is warranted [57]. Intersectoral collaboration will be facilitated if top management supports intersectoral collaboration and heads of departments act as “champions” of such collaboration [33].

### 4.3. Opportunity to Collaborate: There Is No Such Thing as a Free Lunch

The fifth barrier that our study identified was that policy makers had insufficient resources to adjust their policy plans to public health; non-health sectors argued that paying attention to health requires time and money. Due to the budget cuts faced by most municipalities in the Netherlands, both resources are currently in short supply. Additionally, some policy makers argued that they would not approach the regional Public Health Services for advice, as they would have to pay for it. 

This barrier might be overcome if the health policy sectors or the regional Public Health Service were involved in the development of policy plans at an earlier stage, which would help prevent damage having to be repaired afterwards. Health professionals should invest efforts in making these preventable and long-term costs more proactively visible at an earlier stage of the policy cycle (e.g., by conducting health impact assessments [57]). If the regional Public Health Services could decide to offer their policy advice free of charge, the municipal departments might become more proactive in asking for advice, and the non-health sectors might more clearly understand the aims and added value of the intersectoral approach, which was found to be an important facilitator for intersectoral collaboration [33].

The sixth barrier we found was that organizational structures hampered intersectoral collaboration. Switching from a hierarchical to a “flatter” organizational structure [57] may result in policy sectors no longer working in a fragmented system, but being forced to work within intersectoral teams. A “divisionalized adhocracy” is expected to be more suitable for intersectoral collaboration [57], since complex and highly interdependent work fits in better with an organizational structure in which teamwork and liaison managers coordinate work processes, which is thus a prerequisite for the development of integrated public health policies. As Hunter [42] argues, the central feature of all attempts to develop partnerships involving whole systems, rather than individual “silos,” is that they are superimposed on “*a fragmented and largely tribalistic set of arrangements characterized by different cultures and ways of conducting the business*.” Thus, significantly better public health outcomes can be achieved by removing barriers such as sectoral budgets and different priorities and procedures in each sector [33], which can prevent the absorption of significant resources (time, money) that is currently caused by these fundamental errors of organizational structures that have endured for decades [42]. 

### 4.4. Strengths and Limitations

 As with all single-case studies, the results of our study are difficult to generalize, as it involved one municipality and a limited number of governmental actors. In addition, the size of the municipality, which was very small, could also have an effect on the generalizability of our findings. However, most municipalities in the Netherlands are actually small or medium sized (<100,000 inhabitants) [58]. Possible aspects that might be related to the size of the municipality are the strength of social ties and physical proximity (knowing each other professionally and personally, working in the same office), responsibility for more than one policy sector (in smaller municipalities, public health officials are often responsible for one or two other policy sectors as well), the type and magnitude of problems that are encountered (typical urban problems versus local issues), the amount of resources available for public health (lack of resources may function as an incentive for collaboration, while lack of time acts as a discouragement), and the organizational structures (more or less bureaucratic). Another limitation might be the lack of triangulation (e.g., document analysis). A strong point of this study was that we achieved data saturation, and that representatives from all policy disciplines were involved. Another strong point of this study might be that the public health official and the university researcher reflected on each of the interviews together. This enabled the researcher to obtain a more accurate interpretation of the data than would otherwise (without the involvement of someone with background knowledge about the respondents) be possible. The member checks we conducted (a report of each interview was sent to the interviewee) presumably also increased the reliability of our data [59].

## 5. Conclusions

Our single-case-study has identified potentially important facilitators and barriers regarding intersectoral collaboration to promote public health in general. The resources we identified are also applicable to the development of specific integrated public health policies to prevent childhood obesity. This means that public health officials can use this information to anticipate barriers that might hamper intersectoral collaboration for childhood obesity prevention. The most promising facilitating factors we identified were related to motivation, while the least prominent barriers were related to capability, and the most pervasive barriers were related to opportunity. This means that although non-health sectors might be motivated to collaborate with the health sectors, more attention should be paid to the capabilities required, and to create opportunities for collaboration. 

The influence of local government actors and their policies have so far largely been neglected in public health research. Hence, a large potential for developing health promoting policies and interventions by local government organizations still remains to be discovered. Investing in intersectoral collaboration might increase the effectiveness and sustainability of current health promotion efforts to prevent childhood obesity.

## Figures and Tables

**Table 1:** Barriers and facilitators regarding intersectoral collaboration.


Barriers regarding intersectoral collaboration	Reference

*Content-related barriers *	
Lack of awareness of the childhood obesity problem in nonhealth sectors.	Aarts et al. [23]
Limited involvement from other sectors in developing cross-sectoral policies.	Thow et al. [24]
Lack of political support for creating activity-friendly neighborhoods.	Aarts et al. [23]
Neoliberal political climate and individualistic societal climate.	Schwartz and Brownell [25]
Ambiguous political climate, governments do not seem eager to implement restrictive or legislative policy measures since this would mean they have to confront powerful lobbies by private companies.	Nestle [26] Peeler et al. [27] Verduin et al. [28]
Relevance to government's fiscal priorities was important in gaining support for soft drink taxes.	Thow et al. [29]
Lack of agenda-setting: lack of relevance and competing priorities.	Allender et al. [30]
Promoting healthy eating environments is not considered a greater priority for local government than food safety.	Allender et al. [31] Caraher and Coveney [32]
Other legislated planning guidance may take priority for planning and transport professionals.	Bovill [33]
Focusing only on health concerns: not taking into account policy issues of other sectors.	Thow et al. [24]
“Wicked” nature of obesity making it very unattractive to invest in its prevention.	Head [34]
Complexity of the legislative framework.	Allender et al. [35]
Low probability of decreasing the incidence of childhood obesity within the short timeframe that most politicians work in (which is determined by election frequencies).	Aarts et al. [23] Head [34]
Difficulty of developing consensus about ways to tackle the problem due to the lack of hard scientific evidence about effective solutions.	Aarts et al. [23] Head [34] Nishant et al. [36]
Framing of obesity as an individual health problem.	Dorfman and Wallack [37] Klein and Dietz [38] Phillips et al. [39] Merry [40]

*Process-related barriers *	
Local government officials lacking the knowledge and skills to collaborate with actors outside their own department.	Steenbakkers et al. [16]
Insufficient resources (time, budget).	Steenbakkers et al. [16] Woulfe et al. [18] Aarts et al. [23]
Lack of a clear enforcement mechanism.	Thow et al. [24]
Perceived or real lack of power to achieve change.	Thow et al. [29]
Government priorities change.	Nestle [26]
Lack of membership diversity in the collaborative partnerships.	Woulfe et al. [18]
Lack of clarity about the notion of intersectoral collaboration.	Harting et al. [19]
Top-down bureaucracy and hierarchy, disciplinarity and territoriality, sectoral budgets, and different priorities and procedures in each sector.	Bovill [33]
Insufficient organizational structures.	Steenbakkers et al. [16] Woulfe et al. [18] Alter and Hage [41] Hunter [42] Warner and Gould [43]
Poor quality of interpersonal or interorganizational relationships.	Woulfe et al. [18] Isett and Provan [44]
Lack of involvement by managers in collaborative efforts.	Steenbakkers et al. [45]
Lack of communication and insufficient joint planning.	R. Axelsson and S. B. Axelsson [46]
Lack of common vision and leadership.	Woulfe et al. [18] Hunter [42]

Facilitators regarding intersectoral collaboration	

*Content-related facilitators *	
Broad justification for the policy initiative.	Thow et al. [24]
Tailoring of information to the political context: information relevant to the government's agenda.	Schwartz and Brownell [25] Bowen and Zwi [47]
Political risk assessment and saleability.	Schwartz and Brownell [25]
Selection of policy tools that align with the government priorities (e.g., trade commitments)—ideally tools that are already used by trade policy makers in other contexts—and a broad justification for the policy initiative.	Thow et al. [24]

*Process-related facilitators *	
Policy change supported by external funding.	Thow et al. [29]
Cost-benefit analysis for any potential regulatory intervention.	Thow et al. [29]
Systematic evidence base to provide clear feedback on the size and scope of the obesity epidemic at a local level.	Thow et al. [29]
Sensitivity to community and market forces.	Thow et al. [29]
Suitable funding allowing local government to play a part in the promotion of healthy food environments.	Thow et al. [29]
Changing regulations to allow local government to play a part in the promotion of healthy food environments.	Thow et al. [29]
Strategically planning for agenda-setting.	Nestle [26]
Development and implementation of intersectoral health-promoting policies by engaging stakeholders in finance at an early stage to identify priorities and synergies.	Nestle [26]
Developing cross-sectoral advocacy coalitions.	Nestle [26]
Basing proposals on existing legislative mechanisms where possible.	Nestle [26]
Active involvement of health policy makers in initiating the policies.	Nestle [26]
Advocacy making policy uptake and implementation easier.	Thow et al. [24]
Use of existing taxation mechanisms enabling successful policy implementation.	Nestle [26]

**Table 2:** Policy sectors and participants.

Interviewed policy sectors	Participants (*n*): total (8), female (3) male (5)
Youth	(Official 1 (F))
Social affairs	(Official 1 (F))
Tourism	(Official 1 (F))
Municipal environment	(Officials 2 (M) and 3 (M))
Mobility	(Official 4 (M))
Public order and security	(Official 5 (F))
Sports	(Official 6 (M))
Culture	(Official 6 (M))
Education	(Official 6 (M))
Spatial planning	(Officials 7 (F) and 8 (M))

F: female, M: male.

## Appendix

### Items in the Interview protocol

1. Clarifying the role and influence of the policy sectors: their general policies and more specific policies, policy goals.
2. Identifying interfaces between a particular policy sector and the public health sectors.
3. Exploring to what degree a particular policy actor is aware of health aspects within their sector and of the extent to which they are used to collaborating with the regional Public Health Service or the public health department within their own organization.
4. Investigating what the particular policy sector thinks about intersectoral collaboration with the health sector.
5. Exploring opportunities for more collaboration between health and non-health policy sectors.
6. Detecting barriers to attention for public health aspects in non-health policy sectors.
